# Pharmacogenetic prediction of clinical outcome in advanced colorectal cancer patients receiving oxaliplatin/5-fluorouracil as first-line chemotherapy

**DOI:** 10.1038/sj.bjc.6604671

**Published:** 2008-09-16

**Authors:** L Paré, E Marcuello, A Altés, E del Río, L Sedano, J Salazar, A Cortés, A Barnadas, M Baiget

**Affiliations:** 1 Department of Genetics, Hospital de la Santa Creu i Sant Pau, Barcelona 08025, Spain; 2 Department of Clinical Oncology, Hospital de la Santa Creu i Sant Pau, Barcelona 08025, Spain; 3 Department of Hematology, Fundació Althaia, Manresa, Spain; 4 Center for Biomedical Research on Rare Diseases (CIBERER), Barcelona 08025, Spain

**Keywords:** colorectal cancer, oxaliplatin-based chemotherapy, DNA-repair genes, polymorphisms, pharmacogenetics

## Abstract

To determine whether molecular parameters could be partly responsible for resistance or sensitivity to oxaliplatin (OX)-based chemotherapy used as first-line treatment in advanced colorectal cancer (CRC). We studied the usefulness of the excision repair cross-complementing 1 (*ERCC1*), xeroderma pigmentosum group D (*XPD*), *XRCC1* and *GSTP1* polymorphisms as predictors of clinical outcome in these patients. We treated 126 CRC patients with a first-line OX/5-fluorouracil chemotherapeutic regimen. Genetic polymorphisms were determined by real-time PCR on an ABI PRISM 7000, using DNA from peripheral blood. Clinical response (CR), progression-free survival (PFS) and overall survival (OS) were evaluated according to each genotype. In the univariate analysis for CR, *ERCC1*-118 and *XPD* 751 polymorphisms were significant (*P*=0.02 and *P*=0.05, respectively). After adjustment for the most relevant clinical variables, only *ERCC1*-118 retained significance (*P*=0.008). In the univariate analysis for PFS, *ERCC1*-118 and *XPD* 751 were significant (*P*=0.003 and *P*=0.009, respectively). In the multivariant analysis, only the *XPD* 751 was significant for PFS (*P*=0.02). Finally, *ERCC1*-118 and *XPD* 751 polymorphisms were significant in the univariate analysis for OS (*P*=0.006 and *P*=0.015, respectively). Both genetic variables remained significant in the multivariate Cox survival analysis (*P*=0.022 and *P*=0.03). Our data support the hypothesis that enhanced DNA repair diminishes the benefit of platinum-based treatments.

The introduction of oxaliplatin (OX) into treatment regimens for advanced colorectal cancer (CRC) represented a significant advance in the success of chemotherapy. The synergistic effects of 5-fluorouracil (5-FU) and OX have increased response rates by up to 50% in first-line-treated patients (Saunders and Iveson, 2006), allowing surgery of isolated liver metastases that were previously considered unresectable. Oxaliplatin, a platinum-based chemotherapeutic agent that carries a 1,2-diamino-cyclohexane ring, has shown antitumour efficacy *in vitro* and *in vivo*. This bulky carrier group is considered to promote the formation of platinum–DNA adducts. As these adducts are more cytotoxic than those formed from other platinum agents, they are more effective in blocking DNA replication (Raymond *et al*, 1998). These adducts are recognised and repaired by the nucleotide excision repair (NER) pathway, which is a major cellular defence mechanism against the cytotoxic effects of platinum-based chemotherapeutic agents (Reed, 2005). The repairosome responsible for excision involves a number of genes, including excision repair cross-complementing 1 (*ERCC1*). This protein forms a heterodimer with xeroderma pigmentosum group F to execute the incision into the DNA strand, 5′ to the site of damage. There is also a co-ordinate expression of the xeroderma pigmentosum group D (*XPD*) with *ERCC1* and other genes in the NER repairosome complex.

The *ERCC1* gene, on chromosome 19q13.2–q13.3, encodes a protein of 297 amino acids. A number of polymorphisms have been identified in this gene: (i) a common single-nucleotide polymorphism (SNP) at codon 118, position 19007, that causes a C>T change coding for the same amino acid, asparagine; (ii) a second common SNP, located in position 8092 of the 3′-untranslated region, consisting of a C>A change; and (iii) an SNP (G>C) located in intron 3, position 19716. The *XPD* gene (also called *ERCC2*) maps on chromosome 19q13.3 and covers 21.14 kb of genomic DNA. Although several polymorphisms have been reported in this gene, the most frequent is the A751C polymorphism, which causes the substitution of a lysine residue by a glutamine.

Another repair mechanism, known as the base excision repair system (BER), has been identified in human cells. In this system, the XRCC1 protein interacts with DNA ligase III and complexes with DNA polymerase and PARP (polyADP-ribose polymerase), facilitating the repair of DNA strand breaks and several types of DNA damage (Dianov, 2003). The *XRCC1* gene contains 17 exons and is located on chromosome 19q13.2. Although many polymorphisms have been documented, three non-synonymous polymorphisms, Arg194Trp (C>T), Arg280His (G>A) and Arg399Gln (G>A), have been shown to impair DNA-repair capacity in some phenotypic studies.

Glutathione *S*-transferases (GST) are a multigene family of enzymes (cytosolic and membrane bound that catalyse the conjugation of glutathione to electrophilic xenobiotics to inactivate them and facilitate their excretion from the body). Glutathione *S*-transferases have an important function in the metabolism of potentially genotoxic compounds by preventing DNA damage and adduct formation. GSTP1, an isoenzyme belonging to the *π* class, participates in the detoxification of platinum derivatives and is an important mediator of both intrinsic and acquired resistance to platinum (Cullen *et al*, 2003). The gene coding for *GSTP1* is located on chromosome 11q13. The most studied polymorphism is an A>G substitution in position 313 in exon 5 that gives rise to Ile105Val.

In this work, we sought to determine whether different molecular parameters could be responsible in part for resistance or sensitivity to OX-based chemotherapy used as first-line treatment in advanced CRC patients. We studied the usefulness of the *ERCC1*, *XPD*, *XRCC1* and *GSTP1* polymorphisms as predictors of the clinical outcome of these patients.

## Materials and methods

### Patients

One hundred and twenty-six patients diagnosed with metastatic CRC and receiving OX combined with 5-FU as first-line chemotherapy were included in the study. All patients had adequate bone marrow and organ function before treatment. Written informed consent was obtained, and the study was approved by the Institutional Ethics Committee.

### Chemotherapy regimen description

We administered a uniform chemotherapy regimen in this group of patients. It consisted of OX (85 mg m^−2^ infused for 2 h every 2 weeks i.v.) and 5-FU (a pulse dose of 400 mg m^−2^ on days 1 and 2 and a continuous infusion of 1200 mg m^−2^ for 44 h) with i.v. leucovorin. Patients underwent chemotherapy cycles until severe toxicity or disease progression appeared.

### Clinical parameters

Relevant clinical data (gender, age, ECOG, adjuvant therapy, number of chemotherapy cycles, number of metastatic sites, colon *vs* rectum involvement, WBC count, alkaline phosphatase) were obtained from clinical records. We used four baseline clinical parameters (performance status, WBC count, alkaline phosphatase and number of metastatic sites) to classify the patients into three clearly separated risk groups (Kohne *et al*, 2002). The model was adjusted to take this clinical risk classification into account in the multivariate analysis.

We also considered patients with isolated hepatic metastases because chemotherapy in these cases produced a sufficient reduction in tumour size to make curative resection feasible.

Response to treatment and progression-free survival (PFS) and overall survival (OS) were also analysed. Clinical response (CR), which was defined in accordance with WHO criteria (WHO, 1979), was assessed by CT scan every 3 months after the start of chemotherapy. Progression-free survival was defined as the time from the start of chemotherapy to the first occurrence of disease progression or death. Overall survival was calculated from the start of chemotherapy to death regardless of the cause.

Cumulative OX-induced neuropathy was scored on the OX-specific scale reported earlier (Caussanel *et al*, 1990). According to this scale, the grade of cumulative neuropathy depends on the duration and intensity of symptoms (grade 1: paraesthesia, dysesthesia of short duration; grade 2: paraesthesia, dysesthesia persisting between cycles; grade 3: paraesthesia, dysesthesia causing functional impairment).

### Genetic analysis

Genomic DNA was extracted from peripheral leukocytes by the salting-out procedure (Miller *et al*, 1988). All polymorphisms were analysed by means of real-time PCR on an ABI PRISM 7000 Sequence Detection System (Applied Biosystems, Foster City, CA, USA). The TaqMan probes employed are given in Table 1. Each reaction contained template DNA and a final concentration of 1 × TaqMan PCR Master Mix (Applied Biosystems), 300 nM of each primer, 100 nM of wild-type probe (Applied Biosystems) and 100 nM of variant probe (Applied Biosystems). Thermocycling was performed with an initial 50°C incubation for 2 min followed by a 10-min incubation at 95°C. A two-step cycling reaction was performed for 40 cycles, with denaturation at 92°C for 15 s and annealing/extension at 60°C for 1 min. Analysis of the amplification reaction was undertaken using the Sequence Detector software, version 2.0 (Applied Biosystems). Data were analysed using Allelic Discrimination Program (Applied Biosystems). For each polymorphism, a minimum of 10 randomly selected DNA samples were sequenced to confirm the results and were subsequently used as controls.

### Statistical analysis

Differences between categorical variables were measured by the *χ*^2^ test. Logistic regression was used as a multivariate method to ascertain which variables independently predicted CR. Kaplan–Meier estimates and the log-rank tests were employed in the univariate analysis of PFS and OS. A Cox regression model was used for PFS and OS multivariate analyses. The results were considered statistically significant when bilateral *P*-values were less than 0.05. We estimated classical LD measures *D*′ and *r*^2^ with the software LDA (Ding *et al*, 2003).

## Results

The frequencies of the different alleles of the genetic markers analysed (Table 1) showed values (data not shown) similar to those reported in Caucasian populations.

Two SNPs in the *ERCC1* gene, located in intron 3 and exon 4, were in high linkage disequilibrium (*D*′=1, *r*^2^=0.86) (LD: *P*<0.00001): alleles with a C residue in *ERCC1*-118 contained a G residue in position 19716 and alleles with a T in codon 118 carried a C residue in position 19716. As both SNPs gave almost the same information for association analysis, from here on, we consider only the function of *ERCC1*-118.

### Genetic determinants and response

Clinical data are given in Table 2. A total of 126 patients who fulfilled all inclusion criteria were evaluated for response.

Two genetic markers were significantly associated with response: C118T in the *ERCC1* gene (*P*=0.02) and A751C in the *XPD* gene (*P*=0.05). Table 3 shows the relationships between response and these molecular determinants.

The C118T in the *ERCC1* gene maintained its predictive value in a logistic regression model of response (*P*=0.008). The probability of response was 3.7 times greater in patients with a TT or a C/T genotype than in patients with a C/C genotype (CI 95%; relative risk (RR): 1.4–9).

### Genetic determinants and survival

**Figure 1:**
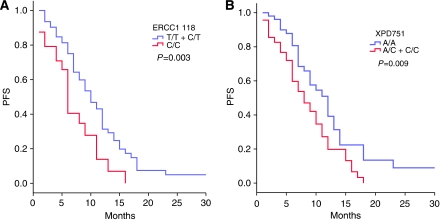
Progression-free survival according to the genotype of (**A**) ERCC1-118 and (**B**) XPD 751.

With regard to PFS, the two variables, the *ERCC1*-118 and the *XPD* 751 SNPs, showed a predictive value. For *ERCC1*-118, the median PFS was 10 months for TT and C/T cases and 6 months for C/C patients (*P*=0.003) (Figure 1A). For *XPD* 751, the median PFS was 12 months for A/A patients and 8 months for A/C and C/C patients (*P*=0.009) (Figure 1B). Only *XPD* 751 and the clinical risk variable remained significant in the multivariant analysis. A/C and C/C patients had a 1.7 × increased risk to progress than A/A patients (RR 1.1–2.8, *P*=0.02).

**Figure 2:**
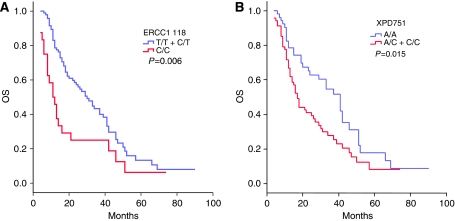
Overall survival according to the genotype of (**A**) ERCC1-118 and (**B**) XPD 751.

As for OS, the *ERCC1*-118 and the *XPD* 751 SNPs defined significantly different survival groups of patients. For *ERCC1*-118, the median OS was 30 months for T/T and C/T and 11 months for C/C cases (*P*=0.006) (Figure 2A). For the *XPD* 751, the median OS was 41 months for AA patients and 17 months for A/C and C/C patients (*P*=0.015) (Figure 2B). Only *ERCC1*-118 and *XPD* 751 SNPs remained significant in the multivariate Cox survival analysis (*P*=0.022; RR=1.8; CI 95%: 1.1–3 and *P*=0.03; RR=1.6; CI 95%: 1.1–2.5, respectively).

### Genetic determinants and neurotoxicity

We analysed whether the *GSTP1* Ile105Val polymorphism was associated with OX-related cumulative neuropathy in this group of patients. A median of 10 cycles per patient (range 6–16) was administered. The median dose intensity of OX was 85 mg m^−2^ per 2 weeks and the median cumulative dose of OX was 850 mg m^−2^ (range 510–1360). Maximal peripheral neuropathy was grade 3 in 4% (five patients), grade 2 in 52.4% (66 patients) and grade 1 in 32.5% (42 patients). No neurotoxicity was detected in the remaining patients. The distribution of the exon 5 *GSTP1* genotype was 35% for the Ile/Ile genotype (44 patients), 49% for the Ile/Val genotype (62 patients) and 16% for the Val/Val genotype (20 patients). Our allele frequency for the Val allele was 0.4 (102 of 252) and resembled earlier reports on allele frequencies for healthy Caucasians and for CRC patients (Watson *et al*, 1998; Welfare *et al*, 1999).

Grade 2 and 3 cumulative peripheral neuropathy was more common in patients with the Ile/Ile genotype (29/44 patients, 66%) than in patients with Ile/Val or Val/Val genotype (42/82 patients, 51%), but the difference did not reach statistical significance (*P*=0.08).

### Genetic determinants and resectability of liver metastases

Sixty-four of 126 patients (51%) had isolated hepatic metastases at diagnosis. Surgery was performed after chemotherapy when there was a sufficient reduction in tumour size (25/64; 39%). Twenty-two of the 25 patients (88%) underwent radical liver surgery with curative intent and three were treated with partial liver surgery. The median OS was 33 months in the group of patients with isolated hepatic metastases and 19 months in the remaining cases (*P*=0.024). The median OS was 46 months in the patients who underwent surgery and 19 months in the remaining cases (*P*=0.001). There was no association between the genetic markers analysed and the probability of receiving surgery after chemotherapy.

## Discussion

Pharmacogenetic data concerning CRC patients treated with platinum-based chemotherapeutic regimens are scarce and sometimes contradictory. In an attempt to obtain clinically useful information, the present discussion is limited to data from similar studies concerning the diagnosis of the patient (CRC patients), the type of treatment (OX/5-FU) and the genetic markers studied (polymorphisms in genes related to the DNA-repair mechanisms). Our results are subsequently discussed gene by gene.

### *ERCC1*

An earlier pharmocogenomic study (Shirota *et al*, 2001) demonstrated that intratumoral ERCC1 mRNA is an independent predictive marker of survival for 5-FU and OX combination chemotherapy. The authors showed a lower gene expression level for the excision repair gene (*ERCC1*) in responders than in non-responders. The same investigators (Park *et al*, 2003, Stoehlmacher *et al*, 2004) devised retrospective studies to determine the association between the *ERCC1* codon 118 and 3′-UTR polymorphism and the clinical outcome of platinum-based chemotherapy in 106 patients with refractory advanced CRC. They found a significant association between the *ERCC1* codon 118 SNP and the clinical outcome: patients with the C/C genotype fared significantly better in terms of OS than those with the thymine allele. There was no significant association between the *ERCC1* C8092A polymorphism and the outcome. In a similar study (with *ERCC1* codon 118 SNP), other authors (Viguier *et al*, 2005) obtained contrasting results. The objective response rate to OX in combination with 5-FU was significantly higher in the T/T genotype group than in the C/T and C/C genotype groups (61.9, 42.3 and 21.4%, respectively; *P*=0.018). In a comprehensive study of common polymorphisms in genes of DNA repair (Moreno *et al*, 2006), it has been demonstrated that patients carrying a C/T and a C/C genotype for the *ERCC1* codon 118 polymorphism show a worse prognosis, with a hazard ratio (HR) of 1.72 (95% CI: 1.04–2.84; *P*=0.036) compared with the T/T cases. The results of a prospective study of 166 patients with advanced CRC treated with first-line Folfox-4 chemotherapy were recently reported. This study, which involved eight medical oncology units from Italy (Ruzzo *et al*, 2007), demonstrated that the *ERCC1*-118 T/T genotype is independently associated with adverse PFS in univariate and multivariate analyses.

The three clinical parameters analysed (CR, PFS and OS) in this work gave consistent results. Our findings demonstrate that patients with the C/C genotype in the *ERCC1* 118 polymorphism have a lower probability of response to treatment and a poorer outcome. The fact that these patients were selected, treated and evaluated by the same clinical researcher, who was unaware of the genotyping findings during the study, confirms the validity of the criteria assessment of response and outcome, and lends further support to our results.

The polymorphism at codon 118 changes a common codon usage (AAC) to an infrequent codon (AAT), both coding for asparagine. Interestingly, it has been proposed that this C>T substitution impairs *ERCC1* translation (Yu *et al*, 1997). In an *in vitro* study, cells carrying the T allele showed a poor capacity to repair the adducts induced by cisplatinum (Yu *et al*, 2000). These data support the pharmacogenetic role of the 118 C>T change and emphasise results that point to the T allele as a marker of a better outcome in patients with CRC treated with OX/5-FU schemes.

### *XPD*

In a study of 106 evaluable patients with advanced CRC who were treated with an OX/5-FU regimen as a second-line treatment, the impact of *XPD* 751 polymorphism was measured in PFS and OS (Stoehlmacher *et al*, 2004). Using the *XPD* 751 Lys/Lys group as a reference, the Gln/Gln group showed a 2.44-fold (95% CI: 1.09–5.44) increased risk of dying, whereas patients with the heterozygous genotype showed an intermediate RR of 1.87 (95% CI: 1.06–3.31) (*P*=0.049), a difference that supported a multivariant analysis (*P*=0.037). The same polymorphism had no impact on PFS. In an earlier study by the same team (Park *et al*, 2001), this polymorphism showed a similar effect on OS in 73 patients. Moreover, 24% of patients with the Lys/Lys genotype responded in contrast to 10% with Lys/Gln and 10% with Gln/Gln genotypes (*P*=0.015). A further study (Ruzzo *et al*, 2007) of 166 patients with similar clinical characteristics indicates that this polymorphism also influenced PFS. Lys/Gln and Gln/Gln patients had HRs of 1.67 (*P*=0.06) and 1.79 (*P*=0.03), respectively, compared with Lys/Lys patients, a difference that was maintained in the multivariant analysis (HR 1.81, *P*=0.04 and HR 2.21, *P*=0.01, respectively). Our results are in line with the above findings. Sixty-seven per cent of our Lys/Lys patients responded compared with 45% of Lys/Gln patients and 40.9% of Gln/Gln patients (*P*=0.047). Likewise, a better OS for Lys/Lys patients was observed in the univariate and multivariate analyses. All the available data, including our present results, indicate that the *XPD* Lys751Gln polymorphism is a useful marker in predicting the clinical outcome of platinum-containing chemotherapy. As the present and previous reports are concordant with the results on the *XPD* 751 variant, a promising role for this marker is proposed.

### *XRCC1*

The first study on the utility of this gene as a pharmacogenetic marker showed that it was predictive of clinical outcome in 61 CRC patients treated with a combination of 130 mg m^−2^ OX and continuous infusion 5-FU (Stoehlmacher *et al*, 2001). In this study, 73% of responders had an Arg/Arg genotype and three were heterozygous, but 66% of non-responders showed a Gln/Gln or Gln/Arg genotype (*P*=0.038). Patients carrying at least one Gln allele had a 5.2-fold increased risk to fail chemotherapy (95% CI: 1.21–22.07). In a subsequent retrospective study that included 106 patients, the same team of investigators failed to confirm *XRCC1* Arg399Gln as a prognosticator (Stoehlmacher *et al*, 2004). Similar negative results were reported in a prospective study of 166 patients where no association was found between this marker and PFS (Ruzzo *et al*, 2007). The results obtained in our study also did not show any evidence of the existence of an association between the three analysed SNPs (Arg194Trp, Arg280His and Arg399Gln) and the clinical parameters that define the CR and survival.

The fact that the NER pathway seems to play a major role in the repair of OX-induced DNA damage may account for the lack of association between clinical outcomes of patients treated with OX-containing regimens and polymorphisms in genes involved in other DNA-repair pathways, as is the case of *XRCC1*, which belongs to the BER system.

### *GSTP1*

In an unpublished report, no association was found between *GST* genotypes and OX-related cumulative neuropathy in a series of patients with advanced CRC (McLeod *et al*, 2003). A recent study (Lecomte *et al*, 2006) investigated the relationship between *GST* polymorphisms and OX-related cumulative neuropathy. In a group of 64 evaluable patients receiving a minimal cumulative dose of 500 mg m^−2^ of OX, the cumulative neuropathy grade 3 was significantly more frequent in patients who were homozygous for the *GSTP1* 105Ile allele than in patients homozygous or heterozygous for the 105Val allele. The authors found no association with the *GSTM1*, *GSTT1* or *GSTP1* exon 6 genotypes. The results of this study suggested that the 105Val allele variant of the *GSTP1* gene confers a significantly decreased risk of developing severe OX-related cumulative neuropathy. We sought to ascertain the existence of this relationship in our group of patients with a comparable cumulative dose of OX. Our findings showed that severe neurotoxicity was less frequent in our group of patients than that reported by Lecomte *et al* (2006). This could be due to the fact that all our patients received first-line treatment, whereas 20% of the patients in Lecomte's series had received a prior chemotherapy treatment. In our patients, as in those in Lecomte's study, severe neurotoxicity was more frequent in the Ile/Ile homozygous patients than in the other groups. Nevertheless, this result did not reach statistical significance, possibly because the number of patients with severe neurotoxicity in our series was small.

The results from this study of patients with advanced CRC treated with first-line OX-based chemotherapy demonstrate the following. (i) A functional polymorphism within the *ERCC1* gene (codon 118) can predict response and OS. (ii) Significant associations exist between the *XPD* Lys751Gln polymorphism and OS and response. As the present and previous reports are concordant with the results on the *XPD* 751 variant, a promising role for this marker is proposed. (iii) No significant association can be found between *GSTP1* Ile105Val and OX-induced neurotoxicity. In conclusion, our data support the hypothesis that there is an inverse relationship between impaired DNA-repair capacity mediated by the NER pathway and superior responses to OX compounds.

## Figures and Tables

**Table 1:** Genetic markers evaluated in this study

**Polymorphism**	**dbSNP**	**NCBI Ref Seq**	**Exon**	**Genotype**	**Amino-acid substitution/position**
*ERCC1* Asn118Asn	rs11615	NM_001983	4	C/T	Asn/118
*ERCC1* G19716C	rs3212948	NM_001983	Intron 3	G/C	—
*ERCC1* C8092A	rs3212986	NM_001983	3′-UTR	C/A	—
*XPD* Lys751Gln	rs13181	NM_000400	23	A/C	Gln/751
*GSTP1* Ile105Val	rs1695	NM_000852	5	A/G	Val/105
*XRCC1* Arg194Trp	rs1799782	NM_006297	6	C/T	Trp/194
*XRCC1* Arg280His	rs25489	NM_006297	9	G/A	His/280
*XRCC1* Arg399Gln	rs25487	NM_006297	10	G/A	Gln/399

**Table 2:** Baseline characteristics of the 126 patients

Gender (men/women)	81 (64%)/45 (36%)
Median age (range, years)	66 (34–83)
	
*Performance status*	
0–1	101 (80%)
>1	25 (20%)
	
*Adjuvant therapy*	
No	89 (71%)
chemotherapy	24 (19%)
chemotherapy+radiotherapy	13 (10%)
	
*Number of cycles of chemotherapy*	
Median (range)	9 (1–20)
	
*Number of patients with isolated hepatic metastases*	
Yes	64 (51%)
No	62 (49%)
	
*Number of patients receiving surgery after QT*	
Yes	25 (20%)
No	39 (80%)
	
*Primary tumour localisation*	
Colon	45 (36%)
Rectum–sigma	81 (64%)
	
*Alkaline phosphatase levels*	
<300 U l^−1^	105 (83%)
⩾300 U l^−1^	21 (17%)
	
*WBC count*	
<10 × 109 per l	113 (90%)
⩾10 × 109 per l	13 (10%)
	
*Risk classification*	
High risk	21 (17%)
Intermediate risk	38 (30%)
Low risk	67 (53%)

**Table 3:** Positive relationships between response and molecular determinants

	**No clinical response**	**Clinical response**	***P***
*ERCC1*-118			0.02
TT	16 (38%)	26 (62%)	
CT	21 (40%)	31 (60%)	
CC	17 (71%)	7 (29%)	
			
*ERCC1*-19716			0.04
G/G	14 (70%)	6 (30%)	
C/G	23 (40%)	35 (60%)	
C/C	18 (38%)	29 (62%)	
			
*XPD* 751			0.05
AA	17 (33%)	35 (67%)	
AC	23 (51%)	22 (50%)	
CC	14 (58%)	10 (42%)	
