# Pharmacogenetic prediction of clinical outcome in advanced colorectal cancer patients receiving oxaliplatin/5-fluorouracil as first-line chemotherapy

**DOI:** 10.1038/sj.bjc.6605004

**Published:** 2009-04-14

**Authors:** L Paré, E Marcuello, A Altés, E del Río, L Sedano, J Salazar, A Cortés, A Barnadas, M Baiget

**Correction to**: British Journal of Cancer (2008) **99**, 1050–1055; doi:10.1038/sj.bjc.6604671

Upon publication of the above manuscript in Volume 99 of *BJC*, the authors noted a small error in the affiliations of L Paré, E del Rio, A Cortés and M Baiget. The full affiliations (addition in bold) is now shown below.

L Paré^1^, E Marcuello^2^, A Altés^3^, E del Río^1,4^, L Sedano^4^, J Salazar^4^, A Cortés^1^, A Barnadas^2^ and M Baiget^1,4^

^1^Department of Genetics, Hospital de la Santa Creu i Sant Pau, **Universitat Autonoma de Barcelona**, Barcelona 08025, Spain; ^2^Department of Clinical Oncology, Hospital de la Santa Creu i Sant Pau, Barcelona 08025, Spain; ^3^Department of Hematology, Fundació Althaia, Manresa, Spain; ^4^Center for Biomedical Research on Rare Diseases (CIBERER), Barcelona 08025, Spain

